# Impact of Nadi Shuddhi Pranayama on Apnea Hypopnea Index, Respiratory Distress Index, and Oxygen Desaturation Index Among Adolescents With Sleep Apnea Syndrome

**DOI:** 10.7759/cureus.65848

**Published:** 2024-07-31

**Authors:** Pallavi Yelkur, Rajajeyakumar Manivel, Syed Mohammed, Shreenivas Rachakonda, Navin Umapathy, V C Akash

**Affiliations:** 1 Pediatrics, Saveetha Medical College and Hospital, Saveetha Institute of Medical and Technical Sciences, Saveetha University, Chennai, IND; 2 Physiology, Saveetha Medical College and Hospital, Saveetha Institute of Medical and Technical Sciences, Saveetha University, Chennai, IND

**Keywords:** sleep apnea syndrome, heart rate variability, apnea–hypopnea index, oxygen desaturation index, respiratory distress index, nadi shodhana pranayama

## Abstract

Background

Sleep apnea syndrome has gained significant recognition over the last decade as a potential cause of substantial childhood morbidity. This study aimed to evaluate the impact of the Apnea Hypopnea Index (AHI), Respiratory Distress Index (RDI), and Oxygen Desaturation Index (ODI) among adolescents with sleep apnea syndrome.

Objectives

The objective of this study is to compare the impact of Nadi Shuddhi Pranayama with routine treatment modalities on the AHI, RDI, and ODI among adolescents with sleep apnea syndrome. Specifically, the study aims to evaluate the effectiveness of Nadi Shuddhi Pranayama in improving AHI, RDI, and ODI in this population.

Methods

This study was an interventional case-control study and enrolled 34 adolescents aged 10 to 18 years who were diagnosed with sleep apnea syndrome. The participants were divided into two groups: the Nadi Shuddhi Pranayama group (n=17) and the control group (n=17). Baseline assessments included the collection of demographic information, medical history, and polysomnography results to determine the AHI, RDI, and ODI. Follow-up assessments were conducted post-intervention to measure changes in the AHI, RDI, and ODI.

Results

There were no significant differences between groups in terms of age (p = 0.176) or gender distribution (p = 0.300). After the intervention period, participants in the Nadi Shuddhi Pranayama group showed significant improvements in the Apnea Hypopnea Index compared to the standard care group (p = 0.007). However, there were no significant differences in the Respiratory Distress Index (p = 0.547) or Oxygen Desaturation Index (p = 0.304) between the groups post-intervention. Subgroup analysis based on severity categories (Mild, Normal) for the Apnea Hypopnea Index showed a significant difference between the Nadi Shuddhi Pranayama and standard care groups (p = 0.037). However, there were no significant differences in the Respiratory Distress Index (p = 0.300) and Oxygen Desaturation Index (p = 0.169) between the groups in this subgroup.

Conclusion

These findings suggest that Nadi Shuddhi Pranayama may improve the Apnea Hypopnea Index in adolescents with sleep apnea, particularly in those with mild symptoms, but did not significantly affect the Respiratory Distress Index or Oxygen Desaturation Index.

## Introduction

Sleep apnea syndrome is defined by episodes of either complete cessation or shallow breathing during sleep caused by partial or total collapse of the upper airway. These episodes typically manifest with snoring, oxygen desaturations, and brief awakenings from sleep [1]. The disorder affects individuals of all age groups, including children and adolescents, and can have significant implications for health, including cognitive function, behaviour, cardiovascular health, and overall quality of life [2]. Among the various treatment modalities available, including continuous positive airway pressure (CPAP) and surgical interventions, complementary therapies such as yoga and pranayama (controlled breathing exercises) have gained attention for their potential benefits in managing sleep-related breathing disorders [1,3,4]. Nadi Shuddhi Pranayama, a form of yogic breathing technique, has been suggested to improve respiratory function and mitigate the severity of sleep apnea through its emphasis on deep, controlled breathing [4].

The practice of pranayama stretches the lung tissue, generating inhibitory signals through actions on slowly adapting receptors and hyperpolarizing currents. These signals from the cardiorespiratory region are thought to synchronize neural elements in the brain, leading to changes in the autonomic nervous system [3,4]. Based on the above-mentioned evidence, we conducted a study on the influence of Nadi Shuddhi Pranayama on the Apnea Hypopnea Index (AHI), Respiratory Distress Index (RDI), and Oxygen Desaturation Index (ODI) among children aged 10 to 18 years with sleep apnea syndrome.

## Materials and methods

This study was an interventional case-control study conducted among adolescents aged 10 to 18 years with sleep apnea syndrome from sleep clinics and paediatric care centres.

Sample size

A total of 34 participants were included in the study and divided into the Nadi Shuddhi Pranayama and control groups. Baseline data were collected, including demographic information, medical history, and polysomnography results, to determine the Apnea Hypopnea Index (AHI), Respiratory Distress Index (RDI), and Oxygen Desaturation Index (ODI). These key parameters were used to assess sleep apnea syndrome. The Apnea Hypopnea Index was calculated by dividing the total number of apneas (complete cessations of airflow) and hypopneas (partial reductions in airflow) by the total hours of sleep, reflecting the severity of sleep apnea. The Respiratory Distress Index, which includes apneas, hypopneas, and additional respiratory disturbances like respiratory effort-related arousals (RERAs), is also divided by the total hours of sleep. The Oxygen Desaturation Index measures how many times per hour of sleep the blood’s oxygen level drops by a certain percentage from the baseline, typically calculated by dividing the number of such desaturation events by the total hours of sleep. These indices are derived from polysomnography, which monitors various physiological variables during sleep. The study was conducted in the Paediatric Department of Saveetha Medical College and Hospital, from June 2023 to January 2024 over a period of six months.

Study design

This study was designed as an interventional case-control study. Participants were randomly assigned to either the intervention group or the control group using computer-generated randomization. The intervention group received Nadi Shuddhi Pranayama training, which involves specific breathing exercises aimed at improving respiratory function and reducing stress. The technique focuses on rhythmic inhalation and exhalation through alternate nostrils to enhance overall well-being and respiratory efficiency. The control group received standard care without any additional interventions. A brief discussion on the technique highlights its traditional use in yoga for balancing the body’s energy and improving respiratory control, which may potentially benefit individuals with sleep apnea.

Methodology

The sleep study was conducted using polysomnography (PSG) over a 6-8 hour period during the night in a sleep lab. The sleep monitor recorded various parameters, including respiratory movements of the chest and abdomen, oxygen saturation and heart rate by oximetry, respiratory airflow by pressure transducer, snoring, body movement, and body position. An adequate overnight sleep study was defined as having a total sleep time of more than 6 hours. Overnight laboratory diagnostic polysomnography was conducted and scored by sleep technologists and interpreted by a pediatric sleep medicine physician according to the American Academy of Sleep Medicine criteria. The polysomnogram parameters examined included the total sleep apnea hypopnea index (AHI), obstructive apnea-hypopnea index (OAHI), oxygen desaturation index (ODI), respiratory distress index (RDI) and oxygen saturation nadir during sleep [5]. Obstructive sleep apnea severity was classified as normal (apnea hypopnea index < 1.5), mild (apnea hypopnea index 1.5 to ≤ 5), and moderate/severe (apnea hypopnea index > 5 events/hour). Specifically, an apnea hypopnea index of 5 to 10 events/hour indicated moderate obstructive sleep apnea, while an apnea hypopnea index ≥ 10 events/hour indicated severe obstructive sleep apnea; these groups were combined as they are more likely to require treatment, such as positive airway pressure therapy, while mild obstructive sleep apnea may warrant observation or watchful waiting. Other causes of sleepiness, including insomnia, delayed sleep phase disorder, and lifestyle factors such as irregular sleep schedules and excessive screen time, as well as psychological stress and poor sleep hygiene, were also evaluated. Oxygen desaturation index is an alternative parameter in screening patients with severe obstructive sleep apnea [6]. Polysomnography reports were analyzed by independent consultants in the ENT department, and various anthropometric measurements were taken to analyze the children.

Nadi Shuddhi Pranayama procedure begins by inhaling through the left nostril while gently closing the right nostril with the thumb. Next, the thumb is released, and the ring finger is used to close the left nostril, allowing the exhale to be released through the right side. Participants in the Nadi Shuddhi Pranayama group underwent regular supervised sessions of Nadi Shuddhi Pranayama, while those in the control group received standard care for sleep apnea syndrome. Follow-up assessments were conducted post-intervention to measure changes in the Apnea Hypopnea Index (AHI), Respiratory Distress Index (RDI), and Oxygen Desaturation Index (ODI). The patients with obstructive sleep apnea who engaged in exercise program showed substantial improvements in the severity of their sleep apnea, as well as enhanced cardiovascular health markers and physical fitness [7,8].

Inclusion criteria

The study included adolescents aged 10-18 years who were diagnosed with obstructive sleep apnea syndrome and had a baseline Apnea Hypopnea Index (AHI) of ≥ 1.5 events per hour. Participants needed to demonstrate a willingness and ability to undergo polysomnography (PSG) in a sleep lab for a minimum of 6 hours. Written informed consent was required from both the participants and their guardians. Additionally, participants had to be able to adhere to the study protocols, which included regular supervised sessions of Nadi Shuddhi Pranayama or standard care for sleep apnea syndrome.

Exclusion criteria

-Adolescents with severe medical conditions affecting sleep or breathing, such as severe asthma, neuromuscular disorders, or heart conditions, were excluded from the study. -Participants currently using medications that affect sleep or breathing, like sedatives or respiratory stimulants, were not included. -Those who had surgery for sleep apnea, such as adenotonsillectomy, within the past year were excluded. -Adolescents with severe mental health issues or cognitive impairments that could make it difficult for them to follow the study rules were not included. -Participants who were unable or unwilling to complete follow-up assessments after the intervention were excluded. -Those who had regularly practiced Pranayama or similar breathing exercises in the past six months were excluded to prevent any effects from their prior experience.

Statistical analysis

Descriptive statistics were used to summarize the baseline characteristics of the participants in both the groups. The primary analysis involved comparing the Apnea Hypopnea Index (AHI), Respiratory Distress Index (RDI), and Oxygen Desaturation Index (ODI) between the Nadi Shuddhi Pranayama and control groups using appropriate statistical tests (e.g., t-tests or Mann-Whitney U tests), depending on the distribution of the data. Subgroup analysis and regression models were used to explore potential confounding factors such as age, sex, and severity of sleep apnea.

## Results

In Table 1, the study included 34 children diagnosed with sleep apnea syndrome, with 17 participants in the Nadi Shuddhi Pranayama group and 17 in the standard care group. At baseline, there were no significant differences between the two groups in terms of age (p = 0.176).

**Table 1:** Comparison of age between Nadi Shuddhi Pranayama group and Standard Care group

	Group		P-value
	Nadi Shuddhi Pranayama (Mean ± SD)	Standard care (Mean ± SD)	
Age (years)	14.35 ± 1.77	15.06 ± 1.14	0.176

Table 2 details the gender distribution between the Nadi Shuddhi Pranayama Group and the Standard Care Group. In the Nadi Shuddhi Pranayama group, 52.9% of participants are female and 47.1% are male. Conversely, the Standard Care group consists of 35.3% female and 64.7% male participants. The p-value for gender distribution between the two groups is 0.3, suggesting no statistically significant difference in gender distribution between the Nadi Shuddhi Pranayama and Standard Care groups.

**Table 2:** Gender distribution between Nadi Shuddhi Pranayama group and Standard Care group

	Group					P-value
		Nadi Shuddhi Pranayama		Standard care		
		Count (N)	Percentage (%)	Count (N)	Percentage (%)	
Gender	Female	9	(52.9%)	6	(35.3%)	0.3
	Male	8	(47.1%)	11	(64.7%)	

In Table 3, pre-intervention polysomnography outcomes indicated that the baseline Apnea Hypopnea Index, Respiratory Distress Index and Oxygen Desaturation Index were comparable between the Nadi Shuddhi Pranayama and Standard Care groups. No statistically significant differences were observed in the Apnea Hypopnea Index, Respiratory Distress Index and Oxygen Desaturation Index.

**Table 3:** Pre-intervention polysomnography outcome among study participants

Pre-intervention	Group		P-value
	Nadi Shuddhi Pranayama (Mean ± SD)	Standard Care (Mean ± SD)	
Apnea hypopnea index	4.82 ± 4.02	6.73 ± 3.44	0.14
Respiratory distress index	14 ± 4.44	14.29 ± 5.36	0.863
Oxygen desaturation index	12.24 ± 5.19	13.88 ± 5.15	0.36

In Table 4, furthermore, subgroup analysis based on severity categories (Mild, Moderate, Severe) for Apnea Hypopnea Index, Respiratory Distress Index and Oxygen Desaturation Index also showed no significant differences between the two groups.

**Table 4:** Distribution of pre-intervention polysomnography severity categories among study participants

Pre-intervention		Group				P-value
		Nadi Shuddhi Pranayama		Standard Care		
		Count (N)	Percentage (%)	Count (N)	Percentage (%)	
Apnea hypopnea index	Mild	11	(64.7%)	5	(29.4%)	0.09
	Moderate	4	(23.5%)	10	(58.8%)	
	Severe	2	(11.8%)	2	(11.8%)	
Respiratory distress index	Mild	11	(64.7%)	10	(58.8%)	0.724
	Moderate	6	(35.3%)	7	(41.2%)	
Oxygen desaturation index	Mild	11	(64.7%)	11	(64.7%)	1
	Moderate	6	(35.3%)	6	(35.3%)	

In Table 5, following the intervention period, participants in the Nadi Shuddhi Pranayama group showed significant improvements in Apnea Hypopnea Index (AHI) compared to the standard care group (p = 0.007). However, no significant differences were observed in the Respiratory Distress Index (p = 0.547) or Oxygen Desaturation Index (p = 0.304) between the two groups post-intervention.

**Table 5:** Post-intervention polysomnography outcome among study participants

Post-intervention	Group		P-value
	Nadi Shuddhi Pranayama (Mean ± SD)	Standard Care (Mean ± SD)	
Apnea hypopnea index	0.82±1.19	2.18±1.55	0.007**
Respiratory distress index	6.94 ±4.31	7.88 ±4.69	0.547
Oxygen desaturation index	6.29±4.18	7.82±4.35	0.304

In Table 6, the post-intervention results comparing the effects of Nadi Shuddhi Pranayama with standard care on the Apnea Hypopnea Index (AHI), Respiratory Distress Index (RDI), and Oxygen Desaturation Index (ODI) reveal some notable differences. For the Apnea Hypopnea Index, the Nadi Shuddhi Pranayama group showed a significant improvement, with 58.8% of participants reaching a normal index compared to only 23.5% in the standard care group, reflected by a P-value of 0.037, indicating statistical significance. In terms of the Respiratory Distress Index, while the Nadi Shuddhi Pranayama group had a higher percentage of participants (52.9%) with a normal index compared to the standard care group (35.3%), the difference was not statistically significant with a P-value of 0.3. Similarly, for the Oxygen Desaturation Index, 58.8% of the Nadi Shuddhi Pranayama group reached normal levels versus 35.3% in the standard care group, but this difference also did not reach statistical significance, as indicated by a P-value of 0.169. Overall, these results suggest that Nadi Shuddhi Pranayama may be particularly effective in improving the Apnea Hypopnea Index, but its effects on the Respiratory Distress Index and Oxygen Desaturation Index require further investigation.

**Table 6:** Distribution of post-intervention polysomnography severity categories among study participants

Post-intervention		Group				P-value
		Nadi Shuddhi Pranayama		Standard Care		
		Count (N)	Percentage (%)	Count (N)	Percentage (%)	
Apnea hypopnea index	Mild	7	(41.2%)	13	(76.5%)	0.037**
	Normal	10	(58.8%)	4	(23.5%)	
Respiratory distress index	Mild	8	(47.1%)	11	(64.7%)	0.3
	Normal	9	(52.9%)	6	(35.3%)	
Oxygen desaturation index	Mild	7	(41.2%)	11	(64.7%)	0.169
	Normal	10	(58.8%)	6	(35.3%)	

## Discussion

The present study was designed to determine the impact of Nadi Shuddhi Pranayama on the Apnea Hypopnea Index, Respiratory Distress Index, and Oxygen Desaturation Index among 10 to 18-year-old children with Sleep apnea syndrome. Nadi Shuddhi Pranayama is a yogic form of breathing exercise that involves the prolongation and control of breath, enhancing conscious awareness, and reshaping breathing habits and patterns. Pulmonary function tests are commonly used to evaluate various aspects of the respiratory system and detect abnormalities [4]. There are studies indicating that pranayama alters various inspiratory and expiratory lung reflexes and interacts with central neural elements to promote new homeostasis in the body and mind [9-11]. The prevalence of complications in children with sleep apnea syndrome is increasing worldwide. Yoga practices, including physical posture, controlled breathing, and deep relaxation, promote overall health. Regular yoga practice has been shown to effectively reduce symptoms of sleep apnea and is considered an alternative therapy for managing children with sleep apnea syndrome [4,11,12].

In the present study, children with sleep apnea syndrome engaged in yogic practice (Nadi Shuddhi Pranayama), which may have increased their energy expenditure. During yoga practice, the lungs and chest expand and contract to their fullest capacity, engaging the muscles to their maximum extent. This maximum inflation and deflation during breathing exercises serve as an important physiological stimulus, releasing surfactants and prostaglandins into the alveolar spaces and significantly increasing lung compliance [3,13]. This study suggests that after yoga training, participants may be able to inhale a greater volume of air into their lungs, thereby increasing the amount of oxygen available to the body. This could potentially help extend the breath-holding duration and decrease respiratory rate. Breathing is an autonomic function that can be consciously controlled, harmonizing the sympathetic and parasympathetic nervous systems [13,14].

The present study demonstrates that regular yogic exercise and Pranayama significantly improved respiratory parameters, especially in patients with mild and normal Apnea Hypopnea Index. Sivapriya et al. demonstrated that the positive results found in the present study can be applied across all schools to improve the pulmonary function of students. A few minutes of daily practice may enhance concentration in school work and studies. Regular practice could contribute to better physical and mental health, potentially leading to a brighter future [4].

A randomized, comparative study conducted by Upadhyay et al. demonstrated that Nadi Shodhana Pranayama showed a significant decrease in auditory reaction time (ART) (p = 0.01), and Bhramari Pranayama showed a highly significant decrease in auditory reaction time (ART) (p < 0.00001). However, there was no statistically significant difference in heart rate variability (HRV) parameters between the groups. The study concluded that both Nadi Shodhana and Bhramari Pranayama can effectively balance sympathovagal tone. Therefore, these pranayama techniques can be practised for the management of essential hypertension [9].

Saisupriya et al. demonstrated a significant decrease in mean heart rate (HR) (p < 0.0001) and an increase in mean respiratory rate (RR), NN50 (p < 0.0001), RMSSD (Root Mean Square of the Successive Differences), and pNN50 (p < 0.0001) following the intervention (Nadi Shodhana pranayama) compared to both pre-intervention values and the control group. These findings suggest that Nadi Shodhana pranayama has immediate effects on inducing parasympathetic activity by reducing respiratory rate and heart rate, highlighting its efficacy in enhancing parasympathetic tone compared to breath awareness alone [15].

Shankarappa et al. investigated the short-term effects of Pranayama on lung parameters in 50 young adults. This study demonstrated significant improvements in lung function parameters, including Forced Vital Capacity (FVC), Forced Expiratory Volume in one second (FEV1), Peak Expiratory Flow Rate (PEFR), Forced Expiratory Flow (FEF), and Breath-Holding Time (BHT). These findings indicate that regular practice of pranayama led to a statistically significant increase in all measured lung parameters among young adults [16]. Pranayama, as a type of yogic breathing exercise, appears to be an effective tool for enhancing lung function and may have therapeutic potential in treating various diseases such as sleep apnea syndrome. Further studies are needed to comprehensively assess other health parameters affected by Pranayama and to explore yoga interventions specifically designed to address changes in sleep apnea syndrome parameters.

Limitations of the study include, the small sample size of 34 participants may limit the generalizability of the results, as a larger sample size would provide more robust data and improve the statistical power of the study. Additionally, the study duration was relatively short, and the long-term effects of Nadi Shuddhi Pranayama on sleep apnea syndrome were not assessed. Future research should consider extended follow-up periods to determine the sustained impact of this intervention. Furthermore, the study relied on self-reported adherence to the Nadi Shuddhi Pranayama practice, which could introduce bias. Objective measures of adherence would help verify the actual practice time and its correlation with the observed outcomes. The study also did not control for potential confounding factors such as diet, physical activity, and other lifestyle variables that could influence respiratory and sleep outcomes.

## Conclusions

The findings of this study suggest that Nadi Shuddhi Pranayama, a form of yogic breathing exercise, may offer significant clinical benefits as adjunctive therapy for managing sleep apnea in adolescents. This study provides promising evidence of the potential efficacy of Nadi Shuddhi Pranayama in improving respiratory outcomes in this population. Furthermore, these findings have implications for the development of holistic interventions and may inform health policies targeting respiratory disorders in children. This study may also guide further research into alternative therapies for sleep apnea and encourage the exploration of complementary practices in pediatric healthcare.
